# Web-Based Patient Self-Reported Outcome After Radiotherapy in Adolescents and Young Adults With Cancer: Survey on Acceptance of Digital Tools

**DOI:** 10.2196/19727

**Published:** 2021-01-11

**Authors:** Marco M E Vogel, Kerstin A Eitz, Stephanie E Combs

**Affiliations:** 1 Department of Radiation Oncology Klinikum rechts der Isar Technical University of Munich Munich Germany; 2 Institute of Radiation Medicine Helmholtz Zentrum München Neuherberg Germany; 3 Deutsches Konsortium für Translationale Krebsforschung DKTK Partner Site Munich Munich Germany

**Keywords:** mHealth, eHealth, young adults

## Abstract

**Background:**

eHealth and mobile health (mHealth) are an evolving trend in the medical field. The acceptance of digital tools is high, and the need is growing.

**Objective:**

Young adults (18-40 years) confronted with a cancer diagnosis present unique needs and require special care. They often have a strong affinity and are familiar with modern technology. On that account, we implemented a web-based symptom and quality of life (QoL) assessment to address patients’ attitudes and willingness to use mHealth tools. The study also aims to evaluate sociodemographic parameters that could influence patients’ opinions.

**Methods:**

A total of 380 young patients aged 18-40 treated with radiotherapy between 2002 and 2017 were included in the trial. We assessed QoL via the European Organization for Research and Treatment of Cancer-Core 30 (EORTC C30) questionnaire and added general questions about mHealth technology. The added questions inquired patients’ opinions regarding general aspects, including technical advances in medicine, mobile and app assistance during cancer treatment, data transfer, and app-specific features. The survey was conducted for 12 months. Participation was voluntary and pseudonymized; prior written consent was obtained.

**Results:**

We achieved a participation rate of 57.6% (219/380) and a completion rate of 50.2% (110/219). The median age was 33 years (range 18-40). Of all participants, 89.1% (98/110) considered new technologies in medicine as positive; 10.9% (12/110) answered with neutral. Nearly all patients (96.4%, 106/110) stated that they would send further data via a web-based platform. Of all, 96.4% (106/110) considered the provided pseudonymization of their data as safe. We further asked the patients if they would use a mobile app for symptom and QoL assessment similar to the present web-based system: 74.5% (82/110) answered with yes and 25.5% (28/110) said they would not use a mobile app in the future. We tested the willingness to use an app on several sociodemographic parameters, such as age, gender, education, health insurance status, and cancer-related parameters: tumor stage, time since radiation treatment, and treatment intention. None of these parameters correlated with app use in this group of young adults. Patients who were generally positive regarding using an app rated several possible functions of a future app. The 3 most requested features were appointment reminders (89.0%, 73/82), contact overview of all involved clinics and physicians (87%, 71/82), and making an appointment via app (78%, 64/82).

**Conclusions:**

eHealth and mHealth tools should be available as an integrated part of a comprehensive cancer care approach. It provides automated, thorough documentation of health parameters during therapy and follow-up for doctors, medical staff, and tumor patients to optimize treatment. With this study, we could show that young adults are the ideal patient population to use eHealth/mHealth tools. Such tools offer further digital support and improve the patients’ need for constant QoL during cancer care.

## Introduction

eHealth and mobile health (mHealth) are an evolving trend in the medical field. The World Health Organization (WHO) defines mHealth as “medical and public health practice supported by mobile devices, such as mobile phones, patient monitoring devices, personal digital assistants, and other wireless devices” [1]. Apps for various health areas exist, supporting our everyday life in cases such as diabetes, weight loss, and depression, or tracking our healthy lifestyle with wearables and devices, such as smartwatches, fitness trackers, and blood pressure monitors [2-4]. Therefore, the application of such tools in the oncologic setting should be discussed. Especially, with the recent COVID-19 pandemic, the desire for health tracking of patients with active treatment for cancer is high. The University of Oklahoma initiated a trial evaluating an app which tracks the symptoms (including COVID-19 symptoms) for patients undergoing chemotherapy (NCT04397614). In previous surveys, we showed the positive acceptance of using such tools: 48.5% of the surveyed patients with cancer and 84.3% of the health care professionals (HCPs) support an oncological app complementing treatment [5,6].

Patient-reported outcome (PRO) is an essential tool. PRO is “any report of the status of a patient’s health condition that comes directly from the patient without interpretation of the patient’s response by a clinician or anyone else” [7]. Convincing studies were performed by Basch et al [8] and Denis et al [9], which suggest that regular contact between patient and HCP via eHealth tools also improves overall survival. Furthermore, Broderick et al [10] reported that the performance status assessment can be improved before initiation of oncologic treatment by PRO. In the literature review by Anatchkova et al [11] regarding PRO use, it became apparent that PRO is still not commonly used in clinical practice. It is emphasized that PRO can support many aspects of cancer care, such as treatment management, monitoring treatment outcomes, quality of life (QoL), and patient communication. The U.S. Food and Drug Administration (FDA) defines QoL as “a general concept that implies an evaluation of the impact of all aspects of life on general well-being” [7].

Young adults (age 18-40 years) confronted with a cancer diagnosis present unique needs and require special care [12]. They differ from pediatric or elderly patients in survival outcomes or epidemiology incidence. Younger patients seem to suffer more in their QoL than older patients. Among others, Champion et al [13] evaluated QoL of breast cancer survivors and showed that younger patients (aged ≤45), compared with older patients (aged 55-70), showed a worse index of well-being (*P*<.001) as well as worse scoring in most of the scales (eg, fatigue, sleep, and overall sexual functioning) [13]. It seems comprehensible as young adults are the group of patients that are in the middle of life. Therefore, a cancer diagnosis may disrupt their employment, relationships, social life, fertility, or independence [14,15]. The constant measure of QoL is essential in this particular group. It can improve their needs in terms of cancer treatment and aftercare. It might influence decisions by HCPs, which often underestimate patients’ preferences and support needs.

In previous studies, we investigated the opinion of 375 patients [5] and 108 HCPs [6] in terms of using mHealth tools in cancer care. We showed that younger patients were more open to modern technologies to support their health (*P*=.032, r=–0.12) [5]. Of all, 68.7% believed that an app would be an ideal complement to the standard follow-up [5]. In total, 98% of HCPs found regular QoL assessment essential, and 93.5% supported the idea of using such an app for scientific research [6]. Basch et al [16] investigated the self-monitoring of chemotherapy toxicity. They reported an 85% compliance of patients with cancer for using an online platform. For young patients with cancer, tracking symptoms and information seeking with eHealth/mHealth tools is found to be relevant by several authors [17,18]. Ramsey et al [19] performed a literature review on eHealth/mHealth for pediatric patients with cancer (mean age 21 years or younger at the time of diagnosis; mean age 39 years or younger at the time of intervention) and concluded that such interventions may play a crucial role in improving health outcomes of young patients undergoing cancer treatment [19]. These previous studies suggest the overall demand for eHealth/mHealth tools in oncology, especially for younger patients. Combined with the fact that young adults are digital natives and familiar with modern tools, it makes sense to incorporate such apps into their regular cancer care. On that account, we implemented exemplarily a web-based symptom and QoL assessment to address patients’ attitudes and acceptance of eHealth/mHealth tools. This study also aims to evaluate sociodemographic parameters that could influence patients’ (cohort of young adults in our case) opinions.

## Methods

This publication is part of the FABIUS trial, which was designed as a prospective study within the Department of Radiation Oncology, Klinikum rechts der Isar, Technical University of Munich (TUM). We included a total of 380 young patients aged 18-40 years treated with radiotherapy between 2002 and 2017.

We assessed symptoms and QoL after radiotherapy via the European Organization for Research and Treatment of Cancer-Core 30 (EORTC C30) questionnaire [20] and added 5 general questions about mHealth technology. The questions added to the EORTC C30 questionnaire are appended as an English translation (see Multimedia Appendices 1 and 2 for the Questionnaire [German] and English translation of the additional questions, respectively).

Patients were contacted via postal mail and asked to participate in the study via a web-based survey system (Survio sro). The platform ensured data protection and security (2048-bit SSL security, ISO/IEC 270001 standards, daily backups).

The added questions inquired patients’ opinions about general aspects, including technical advances in medicine, mobile, and app assistance during cancer treatment, data transfer, and app-specific features. The questions were developed explicitly for the purpose of this study; however, some questions were similar to those used in previous studies by Kessel et al [5,21]. In these studies, we investigated the general attitude of patients with cancer toward mHealth in clinical routine [5] and performed a usability test of an in-house app for QoL evaluation [21]. We descriptively compared the results of this survey with our previously published data [5,21].

One question per page was displayed. The questions for symptom and QoL assessment were designed in multiple-choice format with a single answer and forced entry according to the EORTC C30 questionnaire [20]. The added questions regarding mHealth allowed either single answers (Q: 31, 32, 34, 36), multiple answers (Q: 38), or optional free text (Q: 33, 35, 37). Q31 was designed as a 3-scale question (yes–neutral–no). Q32, Q34, and Q36 were designed as polar questions (yes or no questions) with branching logic. These questions followed a free-text question, and it was only displayed if the previous question regarding mHealth was answered with “no” and personal concerns and problems were inquired. If necessary, we explained technical terms in a footnote. Because all questions were designed with forced entries or with optional free text, only completed questionnaires could be submitted by the user and were analyzed. The participant was able to revise answers using a back button.

The survey was conducted for 12 months between January and December 2017, according to the Checklist for Reporting Results of Internet E-Surveys (CHERRIES) guidelines [22]. Participation was voluntary and pseudonymized; prior written consent was obtained. Each patient received a pseudonym via letter and entered it and the answers in the web-based platform. This way, we were able to reidentify the patient and prevent duplicate entries. The Ethics Committee of the Medical Faculty of Technical University of Munich (TUM) approved the nature and content of the study (Ethics vote: 438/16 S).

In this analysis, we report on the results focusing on the questions about mHealth technology; hence, we will not focus on the QoL measures as there are no comparative values.

We calculated the participation rate as the ratio of unique visitors to the survey site and the total number of contacted patients via letter. The completion rate was calculated using the ratio of completed surveys and the number of unique visitors to the survey.

Statistical calculations were performed using SPSS Statistics version 23 (IBM) in a primarily descriptive way. We used the chi-squared test to test the influence of age, gender, education, health insurance status, tumor stage, time since radiation treatment, and treatment intention. A *P* value <.05 was considered significant.

## Results

Of all patients contacted by letter (n=380), we registered 219 unique visitors. Of those, 110 patients submitted the online survey completely. Fifteen patients left the survey incomplete, and 94 never started the survey. Hence, this results in a participation rate of 57.6% (219/380) and a completion rate of 50.2% (110/219). Median age was 33 years (range 18-40 years); gender distribution was 3:2 (female:male). Table 1 presents the complete participants’ characteristics.

**Table 1:** Participants’ characteristics (N=110).

Characteristic		Values
**Gender**		
	Male, n (%)	45 (40.9)
	Female, n (%)	65 (59.1)
**Age (years), median (range)**		33 (18-40)
	18-30, n (%)	37 (33.6)
	30-40, n (%)	73 (66.4)
**Health insurance status**		
	Privately insured, n (%)	17 (15.5)
	State insured, n (%)	93 (84.5)
**Education**		
	High school, n (%)	28 (25.5)
	Above high school, n (%)	50 (45.5)
	University degree, n (%)	24 (21.8)
	Unknown	8 (7.3)
**Tumor entity**		
	Breast cancer and gynecological tumor, n (%)	23 (20.9)
	Prostate cancer and urological tumor, n (%)	4 (3.6)
	Neurooncological tumor, n (%)	31 (28.2)
	Upper and lower gastrointestinal cancer, n (%)	1 (0.9)
	Hematological cancer, n (%)	28 (25.5)
	Skin cancer, n (%)	2 (1.8)
	Head and neck cancer, n (%)	6 (5.5)
	Bone cancer, n (%)	4 (3.6)
	Soft tissue tumor, n (%)	9 (8.2)
	Benign tumor, n (%)	2 (1.8)
**Tumor stage**		
	Advanced/Metastatic, n (%)	48 (44.0)
	Low grade, n (%)	62 (56.4)
**Treatment intention**		
	Curative, n (%)	103 (93.6)
	Palliative, n (%)	7 (6.4)
Time since radiotherapy (months), median (range)		27 (0.2-178)

Of all participants, 89.1% (98/110) considered new technologies in medicine as positive; 10.9% (12/110) answered with neutral. Nearly all patients (96.4%, 106/110) stated that they would send further data via a web-based platform. Of all, 96.4% (106/110) considered the provided pseudonymization of their data as safe. We further asked the patients if they would use a mobile app for symptom and QoL assessment similar to the present web-based system: 74.5% (82/110) answered with yes and 25.5% (28/110) said they would not use a mobile app in the future.

We tested the willingness to use such an app for symptom and QoL assessment on several sociodemographic parameters, such as age, gender, education, health insurance status, and cancer-related parameters: tumor stage, time since radiation treatment, and treatment intention. None of these parameters correlated with the willingness to use an app in this group of young adults (Table 2).

**Table 2:** Evaluation of sociodemographic parameters on the willingness to use an app (according to Pearson chi-square tests).

Parameter	*P* value
Age (18-30 vs 31-40)	.846
Gender (male vs female)	.257
Education (low vs medium vs high)	.413
Health insurance status (private vs state)	.843
Tumor stage (advanced vs low-grade)	.220
Treatment intention (curative vs palliative)	.110
Time since radiotherapy (<24 months vs ≥24 months)	.327

**Figure 1:**
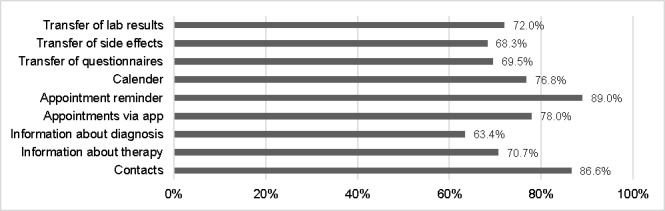
Rating of possible app features by patients willing to use an app (N=82).

The most mentioned reasons against using an app were as follows: smartphones are less safe (7/28), the patient does not want to be reminded of the illness on a smartphone (3/28), no need for one as there is no current treatment (2/28), and not owning a smartphone (2/28). Patients who were generally positive regarding using an app rated several possible functions of a future app (Figure 1). The 3 most requested features were appointment reminders (89%, 73/82), contact overview of all involved clinics and physicians (87%, 71/82), and making an appointment via app (78%, 64/82; Figure 1).

**Figure 2:**
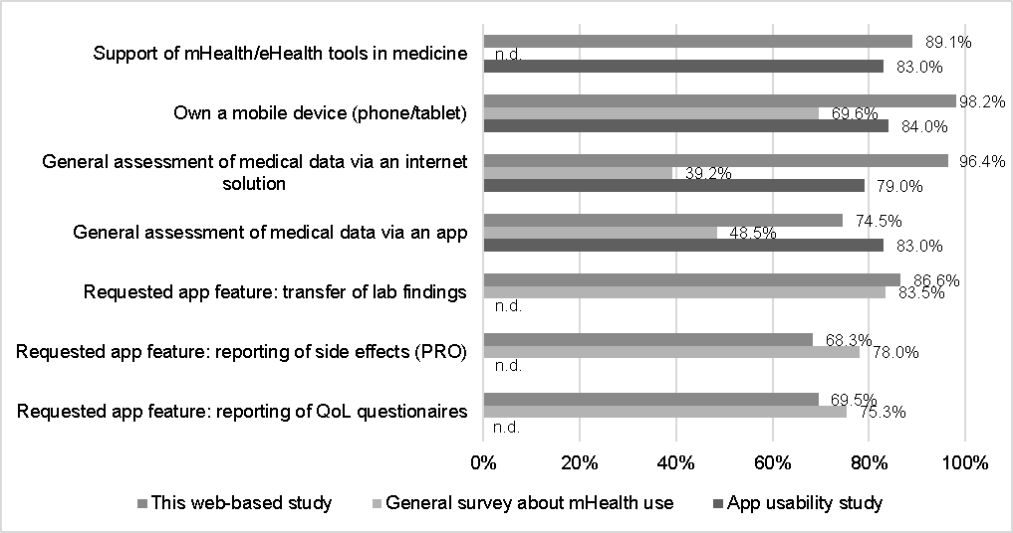
Comparison of results for this web-based study with the previously published results by Kessel et al regarding a general survey about mHealth use [5] and an app usability study [21]. mHealth: mobile health; PRO: patient-reported outcome; QoL: quality of life.

Figure 2 shows the descriptive comparison of questions of this study (median age 33 years; range 18-40 years) with previous surveys by Kessel et al: a general survey of 375 patients with cancer about mHealth use in oncology (median age 59 years; range 18-92 years) [5] and a usability study with 81 patients with cancer on a prototype for an oncologic app (median age 55 years; range 21-80 years) [21]. The surveys were conducted with similar questions as this study.

## Discussion

### Principal Findings

Patients’ compliance with a web-based symptom and QoL assessment depends on their general technical affinity. Our cohort of young adults confirmed that these are the ideal patients to be supported by digital health care as they show high acceptance (96.4%, 106/110). No sociodemographic or cancer-related factors could be found influencing the attitude and willingness to use eHealth/mHealth tools.

The advancing digitalization offers countless possibilities in the health sector: from simple pedometers to complex behavioral therapy for patients with depression. Fitness bracelets, digital blood pressure meter, and blood glucose meters are now connected to the smartphone as a matter of course, and the data are evaluated [2,23,24]. Especially in oncology, due to the many and complex prognostic factors, a broad database including diagnostic, therapy, and regular reported PRO data combined with artificial intelligence–based analyses could have a lasting positive effect on the success of the therapy [25-27].

Previously we showed that young adults are most likely to use modern solutions such as apps and web-based tools to support their cancer treatment [5]. Nearly all patients (96.4%, 106/110) stated that they would send further medical data via a web-based platform. Compared with an app-based assessment, still, 74.5% (82/110) would be willing to use such a tool to support their treatment course. This is comparable to the app usability test we performed on a group of patients with cancer (median age 55; range 21-80 years) [21]. Here 83% would use a web-based and 79% an app-based tool to assess medical data. In a further study where we asked patients with cancer in several departments about their attitude to modern technologies, 39.2% indicated that they would use a web-based and 48.5% an app-based solution [5]. These numbers are relatively smaller as in the other cohorts; however, the survey was conducted without preselecting for age or favorable attitude toward modern technologies. Hence, all the critics and opponents of the idea of implementing an app account for the smaller percentages of acceptance.

In our group of young adults, no sociodemographic or cancer-related factors could be found influencing the attitude and willingness to use mHealth tools. This corresponds well with the high acceptance (89.1%, 98/110) of new technologies in medicine. Certainly, irrespective of whether an app or web-based data transfer is applied, a secure and safe approach must be ensured. Generally, patients understand the concept of pseudonymization and accept it as a safe way of data transmission (96.4%, 106/110). Informing the patient extensively about data management is an important part that should not be underestimated to gain the patients’ trust. Besides, to ensure institutional review board approval and fulfill all legal requirements, the data transfer is the most critical part when implementing an eHealth/mHealth solution into a clinical environment [25]. Still, significant obstacles are the lack of technical standards and often difficulties integrating a system that needs external access to the clinic network [25,28,29].

The 3 most desired features of an app were the possibility of making an appointment via app (78%, 64/82), an appointment reminder (89%, 73/82), and the general possibility to store all contacts of the involved physicians in one place (87%, 71/82). The latter makes much sense, as during complex and interdisciplinary cancer treatment, many HCPs from the treating clinic as well as external care providers (eg, radiologists, oncologists, family physicians) are involved. In a review by Iribarren et al [30], mHealth apps and their activities were investigated. All these features were also named as essential activities. Compared to the results of our previous survey about mHealth [5], the desired app features in this study are equally important to patients of all age groups (Figure 2).

Especially during the time of the COVID-19 virus pandemic, clinicians, especially oncologists, wish for digital/mobile options to contact patients to minimize patient presence while guaranteeing access to treatment and safety of the patients and their families. Patients with cancer are individuals confronted with the most challenging impact as they have acute or chronic medical conditions and often a weakened immune system. With a mobile or web-based connection, it is possible to get regular feedback, such as current health status reported by patients themselves, and decide if a visit, for example, for a chemotherapy session, is possible [31]. During the COVID-19 times, it is evident that the digitalization and implementation of eHealth/mHealth tools are missing and need to be permanently installed in a clinic [32,33].

### Limitations

Our study has some limitations. We sent the study invitation to 380 patients, of which 110 participated and completed the survey. Unfortunately, we have no information about the critics’ and opponents’ attitude to the evaluated web-based QoL assessment and can only present the results of the supporters. We did not subselect patients by tumor type and invited all treated patients between 2002 and 2015. Patients with benign tumors that are no longer in treatment or follow-up might consider themselves as healthy and are most likely not willing to participate in a cancer-related survey.

### Future Directions

In the future, to provide a comprehensive solution of eHealth-/mHealth-supported cancer care, all involved parties must agree to an individual, age-specific approach. This includes a web-based and app-based assessment of medical data and the thorough integration into the clinical environment to connect the patient-reported parameters with all health-relevant data. It must be assured that the used software solutions are professional and validated to guarantee patients’ safety [25,29,34-37]. Because our data are promising, our goal is to implement an app into our day-to-day clinical routine. However, with the first attempts of developing an own app [21], we quickly realized that such projects must be seen in a broader context. We need to develop across-the-board apps with a variety of interfaces for various medical disciplines. Such projects can only be accomplished with strong partners in politics and industry.

### Conclusion

eHealth and mHealth tools should be available as an integrated part of a comprehensive cancer care approach. Such tools provide automated, comprehensive documentation of health parameters during therapy and follow-up care for doctors, medical staff, and tumor patients to optimize treatment. IT departments need to strengthen the implementation and create a comprehensive eHealth solution integrated into the existing IT infrastructure. With the FABIUS trial, we could show that young adults are the ideal patient population to use eHealth/mHealth tools. Such tools offer further digital support and improve the patients’ need for constant QoL during cancer care.

## Appendix

Multimedia Appendix 1 Web-based original questionnaire.

Multimedia Appendix 2 English translation of the additional questions.

## Abbreviations

CHERRIES: Checklist for Reporting Results of Internet E-Surveys
EORTC C30: European Organization for Research and Treatment of Cancer-Core 30
FDA: U.S. Food and Drug Administration
HCP: health care professional
PRO: patient-reported outcome
QoL: quality of life
TUM: Technical University of Munich
WHO: World Health Organization
