# Supplementary material for: Web-Based Patient Self-Reported Outcome After Radiotherapy in Adolescents and Young Adults With Cancer: Survey on Acceptance of Digital Tools
Source: JMIR Mhealth Uhealth. 2021 Jan 11;9(1):e19727. doi: 10.2196/19727 (PMC7834941; doi:10.2196/19727)
Supplement: Multimedia Appendix 1 [file mhealth_v9i1e19727_app1.pdf]

ACHTUNG: Dies ist eine Umfrage Vorschau, eingereichte Antworten werden nicht gespeichert. Klicken Sie hier (https://www.survio.com/survey/d/U9Q1S6Y2E2I6I4E2F), die Umfrage zu ausfüllen.

Patienten-Umfrage – Web-basierte Erfassung von Gesundheitsparametern von Patienten zur Unterstützung der Nachsorge

EORTC QLQ-C30 (Version 3)

Wir sind an einigen Angaben interessiert, die Sie und Ihre Gesundheit betreffen. Bitte beantworten Sie die folgenden Fragen, indem Sie die Antwort anklicken, die am besten auf Sie zutrifft. Es gibt keine „richtigen" oder „falschen" Antworten. Ihre Angaben werden streng vertraulich behandelt.

1

Bitte geben Sie Ihr Pseudonym ein:

Geben Sie hier Ihre Antwort ein.

*Noch **50** Zeichen übrig*

2

Bereitet es Ihnen Schwierigkeiten, sich körperlich anzustrengen (z. B. eine schwere Einkaufstasche oder einen Koffer zu tragen)?

- Überhaupt nicht
- Wenig
- Mäßig
- Sehr

3

Bereitet es Ihnen Schwierigkeiten, einen längeren Spaziergang zu machen?

- Überhaupt nicht
- Wenig
- Mäßig
- Sehr

4

Bereitet es Ihnen Schwierigkeiten, eine kurze Strecke außer Haus zu gehen?

- Überhaupt nicht
- Wenig
- Mäßig
- Sehr

5

Müssen Sie tagsüber im Bett liegen oder in einem Sessel sitzen?

- Überhaupt nicht
- Wenig
- Mäßig
- Sehr

6

Brauchen Sie Hilfe beim Essen, Anziehen, Waschen oder Benutzen der Toilette?

- Überhaupt nicht
- Wenig
- Mäßig
- Sehr

7

Waren Sie bei Ihrer Arbeit oder bei anderen tagtäglichen Beschäftigungen eingeschränkt?

Während der letzten Woche

- Überhaupt nicht

Wenig

Mäßig

Sehr

8

Waren Sie bei Ihren Hobbys oder anderen Freizeitbeschäftigungen eingeschränkt?

Während der letzten Woche

Überhaupt nicht

Wenig

Mäßig

Sehr

9

Waren Sie kurzatmig?

Während der letzten Woche

Überhaupt nicht

Wenig

Mäßig

Sehr

10

Hatten Sie Schmerzen?

Während der letzten Woche

Überhaupt nicht

Wenig

Mäßig

Sehr

11

Mussten Sie sich ausruhen?

Während der letzten Woche

Überhaupt nicht

Wenig

Mäßig

Sehr

12

Hatten Sie Schlafstörungen?

Während der letzten Woche

Überhaupt nicht

Wenig

Mäßig

Sehr

13

Fühlten Sie sich schwach?

Während der letzten Woche

Überhaupt nicht

Wenig

Mäßig

Sehr

14

Hatten Sie Appetitmangel?

Während der letzten Woche

Überhaupt nicht

Wenig

Mäßig

Sehr

15

War Ihnen übel?

Während der letzten Woche

- Überhaupt nicht
- Wenig
- Mäßig
- Sehr

16

Haben Sie erbrochen?

Während der letzten Woche

- Überhaupt nicht
- Wenig
- Mäßig
- Sehr

17

Hatten Sie Verstopfung?

Während der letzten Woche

- Überhaupt nicht
- Wenig
- Mäßig
- Sehr

18

Hatten Sie Durchfall?

Während der letzten Woche

- Überhaupt nicht
- Wenig
- Mäßig
- Sehr

19

Waren Sie müde?

Während der letzten Woche

- Überhaupt nicht
- Wenig
- Mäßig
- Sehr

20

Fühlten Sie sich durch Schmerzen in Ihrem alltäglichen Leben beeinträchtigt?

Während der letzten Woche

- Überhaupt nicht
- Wenig
- Mäßig
- Sehr

21

Hatten Sie Schwierigkeiten, sich auf etwas zu konzentrieren, z. B. auf das Zeitung lesen oder das Fernsehen?

Während der letzten Woche

- Überhaupt nicht
- Wenig
- Mäßig
- Sehr

22

Fühlten Sie sich angespannt?

Während der letzten Woche

- Überhaupt nicht
- Wenig
- Mäßig
- Sehr

23

Haben Sie sich Sorgen gemacht?

Während der letzten Woche

- Überhaupt nicht
- Wenig
- Mäßig
- Sehr

24

Waren Sie reizbar?

Während der letzten Woche

- Überhaupt nicht
- Wenig
- Mäßig
- Sehr

25

Fühlten Sie sich niedergeschlagen?

Während der letzten Woche

- Überhaupt nicht
- Wenig
- Mäßig
- Sehr

26

Hatten Sie Schwierigkeiten, sich an Dinge zu erinnern?

Während der letzten Woche

- Überhaupt nicht
- Wenig
- Mäßig
- Sehr

27

Hat Ihr körperlicher Zustand oder Ihre medizinische Behandlung Ihr Familienleben beeinträchtigt?

Während der letzten Woche

- Überhaupt nicht
- Wenig
- Mäßig
- Sehr

28

Hat Ihr körperlicher Zustand oder Ihre medizinische Behandlung Ihr Zusammensein oder Ihre gemeinsamen Unternehmungen mit anderen Menschen beeinträchtigt?

Während der letzten Woche

- Überhaupt nicht
- Wenig
- Mäßig
- Sehr

29

Hat Ihr körperlicher Zustand oder Ihre medizinische Behandlung für Sie finanzielle Schwierigkeiten mit sich gebracht?

Während der letzten Woche

- Überhaupt nicht
- Wenig
- Mäßig
- Sehr

30

Bitte geben Sie bei den folgenden Fragen die Zahl zwischen 1 und 7 an, die am besten auf Sie zutrifft

	1 sehr schlecht	2	3	4	5	6	7 ausgezeichnet
Wie würden Sie insgesamt Ihren Gesundheitszustand während der letzten Woche einschätzen?							
Wie würden Sie insgesamt Ihre Lebensqualität während der letzten Woche einschätzen?							

31

Neue Technologien in der Medizin: Wie finden Sie diese Entwicklung?

(wie z.B. diese Art einer webbasierten Patienten-Umfrage)

- Finde ich gut
- Ich bin dem gegenüber neutral eingestellt
- Finde ich nicht gut

32

Wären Sie bereit, uns zukünftig auch weitere Daten über eine Web-Anwendung zukommen zu lassen?

- Ja
- Nein

33

Warum würden Sie keine weiteren Daten übertragen?

Geben Sie hier Ihre Antwort ein.

*Noch **250** Zeichen übrig*

34

Ihre Daten werden an uns pseudonymisiert übertragen: Finden Sie diese Art der Datenübertragung sicher?

(Pseudonymisierung: Das bedeutet, dass Ihre Daten nicht direkt Ihrem Namen zugeordnet werden, sondern einem Verschlüsselungscode, den nur Mitwirkende der Studie entschlüsseln können. Alle berechtigen Personen haben eine Verschwiegenheitserklärung unterzeichnet. Damit wird die strenge Vertraulichkeit Ihrer Daten gewährleistet. Die Daten sind vor fremdem Zugriff geschützt.)

- Ja
- Nein

35

Welche Bedenken haben Sie bezüglich pseudonymisierter Datenübertragung?

Geben Sie hier Ihre Antwort ein.

*Noch **1500** Zeichen übrig*

36

Würden Sie eine von uns kostenlos zur Verfügung gestellte App (verfügbar für verschiedene Smartphone-Betriebssysteme) herunterladen und zur Datenübertragung (z.B. von Nebenwirkungen) an uns nutzen?

- Ja

Nein

37

Warum würden Sie eine App nicht nutzen?

Geben Sie hier Ihre Antwort ein.

*Noch **1500** Zeichen übrig*

38

Welche der folgenden Zusatzfunktionen einer solchen App würden Sie nutzen?

(Mehrfachauswahl möglich)

- Übermittlung von Laborwerten an uns als Ihre betreuende Klinik
- Erinnerung an das Ausfüllen und die Übermittlung von aktuellen Nebenwirkungen und Gesundheitsparametern
- Erinnerung an das Ausfüllen und die Übermittlung von Fragebögen (z.B. wie den, den sie soeben ausgefüll haben)
- Terminkalender während und nach der Therapie
- Erinnerung an Termine (z.B. Nachsorgetermine)
- Terminvereinbarung bzw. -anfrage
- Nachschlagewerk und Wissenswertes zu Ihrer Diagnose
- Nachschlagewerk und Wissenswertes zu Ihrer Therapie (Pflegehinweise, Übungen, Wegepläne etc.)
- Kontaktinformationen aller beteiligen Ärzte und Abteilungen
- Kommentare / weitere Vorschläge:

Geben Sie hier Ihre Antwort ein.

*Noch **100** Zeichen übrig*

39

Haben Sie noch Kommentare für uns?

Geben Sie hier Ihre Antwort ein.

*Noch **1500** Zeichen übrig*

ABSENDEN

Kostenlos **Umfrage erstellen** (http://www.survio.com/de/?source=survey_footer&medium=link&term=survey_link) ✓
Powered by Survio (http://www.survio.com/de/merkmale-funktionen?source=survey_footer&medium=link&term=brand)
