# Supplementary material for: Web-Based Patient Self-Reported Outcome After Radiotherapy in Adolescents and Young Adults With Cancer: Survey on Acceptance of Digital Tools
Source: JMIR Mhealth Uhealth. 2021 Jan 11;9(1):e19727. doi: 10.2196/19727 (PMC7834941; doi:10.2196/19727)
Supplement: Multimedia Appendix 2 [file mhealth_v9i1e19727_app2.pdf]

English translation of the additional questions to the EORTC-QLQ-C30 questionnaire:

31. New technologies in medicine: What do you think about this development?

   a. I like it.
   b. I am neutral.
   c. I don’t like it.

32. Would you be willing to provide us with further data via web application in the future?

   a. Yes
   b. No

33. Why wouldn’t you transfer further data?

   a. Free-text response

34. Your data is transferred pseudonymized: Do you find this type of data transfer secure?

(Pseudonymization: This means that your data will not be assigned directly to your name, but to an encryption key that only study personnel can decipher. All authorized personnel have signed a confidentiality agreement. This guarantees the strict confidentiality of your data. The data is protected from unauthorized access).

   a. Yes
   b. No

35. What concerns do you have about pseudonymized data transmission?

   a. Free-text response

36. Would you download an app provided by us free of charge (available for various smartphone operating systems) and use it for data transfer (e.g. of side effects)?

   a. Yes
   b. No

37. Why wouldn't you use such an app?

   a. Free-text response

38. Which of the following additional functions of such an app would you use?

   a. Transmission of laboratory values to your treating clinic
   b. Reminder to fill out and submit current side effects and health parameters

c. Reminder to fill out and submit questionnaires (e.g. like the one you just have filled out)
d. Appointment calendar during and after the therapy
e. Reminder of appointments (e.g. aftercare appointments)
f. Making appointments or appointment requests
g. Things to know about your therapy (care instructions, exercises, route maps, etc.)
h. Contact information of all participating doctors and departments
i. Others: *(free-text response)*
